# Serum Selenium and Age as Predictors of Metabolic Health in Middle-Aged Women: A Regression-Based Study

**DOI:** 10.3390/nu17091429

**Published:** 2025-04-24

**Authors:** Daria Schneider-Matyka, Anna Maria Cybulska, Kamila Rachubińska, Bogumiła Pilarczyk, Mariusz Panczyk, Elżbieta Grochans, Dorota Ćwiek, Iwona Bojar, Jacek Brodowski

**Affiliations:** 1 Department of Nursing, Pomeranian Medical University in Szczecin, Żołnierska Str. 48, 71-210 Szczecin, Poland; daria.schneider.matyka@pum.edu.pl (D.S.-M.); kamila.rachubinska@pum.edu.pl (K.R.); elzbieta.grochans@pum.edu.pl (E.G.); 2 Department of Animal Reproduction Biotechnology and Environmental Hygiene, West Pomeranian University of Technology, Klemensa Janickiego Str. 3. 29, 71-217 Szczecin, Poland; bogumila.pilarczyk@zut.edu.pl; 3 Department of Education and Research in Health Sciences, Faculty of Health Sciences, Medical University of Warsaw, Litewska Str. 14/16, 00-581 Warsaw, Poland; mariusz.panczyk@wum.edu.pl; 4 Department of Obstetrics and Pathology of Pregnancy, Pomeranian Medical University in Szczecin, Żołnierska Str. 48, 71-210 Szczecin, Poland; dorota.cwiek@pum.edu.pl; 5 Department of Women’s Health, Institute of Rural Health in Lublin, Kazimierza Jaczewskiego Str. 2, 20-950 Lublin, Poland; iwonabojar75@gmail.com; 6 Primary Care Department, Pomeranian Medical University in Szczecin, Żołnierska Str. 48, 71-210 Szczecin, Poland; jacek.brodowski@pum.edu.pl

**Keywords:** selenium, lipid metabolism, middle-aged women

## Abstract

**Background:** This study investigated the relationship between serum selenium concentration and metabolic markers—specifically lipid parameters and glycemic control indicators (fasting glucose, insulin, HbA1c, HOMA-IR)—in middle-aged women, considering age as a covariate. **Methods:** A total of 387 women aged 45–67 years participated. Serum levels of selenium, glucose, insulin, HbA1c, HDL, LDL, and triglycerides were measured. Multiple linear regression models were used to evaluate the predictive value of the serum selenium concentration compared to age in predicting lipid and glycemic markers. **Results:** Selenium concentration was significantly and positively associated with HDL cholesterol only. No significant relationships were found between selenium and glycemic markers or other lipid parameters. In contrast, age emerged as a consistent positive predictor of LDL cholesterol, fasting glucose, and HbA1c levels. Furthermore, exploratory analysis suggested that HbA1c may influence the relationship between selenium and HDL/LDL cholesterol, though no formal moderation analysis was performed. **Conclusions:** Although serum selenium concentrations were within the normal range, their predictive value was limited to HDL cholesterol. Age showed a stronger and more consistent association with key metabolic markers, highlighting its importance as a predictor of cardiometabolic health in middle-aged women.

## 1. Introduction

Selenium (Se) is a trace element essential for the synthesis of selenoproteins, which are involved in antioxidant defense systems and affect redox signaling [1]. Due to the antioxidant and anti-inflammatory properties of selenium, numerous studies have assessed the relationship between its levels and disorders and how this is associated with increased oxidative stress and inflammation (cardiovascular and neurodegenerative diseases, diabetes, and cancer) [1,2,3]. Since type 2 diabetes is associated with oxidative stress, selenium was initially expected to have a beneficial effect on diabetic patients due to its antioxidant and anti-inflammatory effects. Consistent with these findings, supplementation with antioxidants, including selenium, has been found to reduce the risk of diabetes [3,4]. Recently, researchers have focused on selenoproteins to analyze the effects of selenium intake in humans. Rayman et al. observed that high serum selenium concentrations accompany type 2 diabetes [5].

An important role of selenium is to participate in the development of significant enzyme proteins. There are three main families of enzymes that have selenium in their structure: glutathione peroxidases (GPxs), iodothyronine deiodinases, and thioredoxin reductases (TrxR). In addition, 12 individual selenoproteins have been detected in the human body, which have a variety of functions [6]. The glutathione peroxidase family includes glutathione pe-roxidase-1 (GPx-1), which is one of the most important enzymes protecting cells against oxidative stress [5]. In addition to the physiological role of GPx-1, experiments with mouse models have shown that its overexpression causes hyperglycemia and hyperinsulinemia. Consistent with this side effect, wild-type animals showed better insulin sensitivity compared to GPx-1 overexpressing mice [7]. The role of selenium in diabetes is still under discussion, including both selenium deficiency and its supply above dietary requirements [8].

The incidence of diabetes is increasing worldwide. In 2019, over 463 million people suffered from diabetes, and the incidence is expected to rise to 700 million by 2045 [9]. More than 90% of diabetic people have type 2 diabetes [10], which involves numerous complications, including cardiovascular disease (CVD), peripheral neuropathy, stroke, infection, chronic renal failure, and retinopathy. All these contribute significantly to the high mortality, morbidity, and socioeconomic burden associated with diabetes [11].

Studies by Nie et al. indicate that selenium plays a role in lipid metabolism, affecting blood cholesterol levels. For example, selenoproteins show anti-inflammatory properties and may protect neuronal microstructures [12]. A study by Ouyang et al. suggests that selenium supplementation can lower insulin and HOMA-IR levels and increase HDL cholesterol levels in patients with cardiometabolic diseases [13]. The results of Faghihi et al.’s study, on the other hand, indicate that selenium supplementation in patients with type 2 diabetes can lead to adverse changes in glucose homeostasis, including increases in HbA1c and HDL cholesterol levels [14]. Mehvari et al. found that patients with type 2 diabetes had higher levels of LDL and triglycerides and lower levels of HDL compared to the control group. Selenium and vitamin E improved the lipid profile, increasing HDL levels and lowering LDL and triglycerides, and increased PON-1 activity, reducing oxidative stress. Lower PON-1 activity in diabetic patients was associated with higher oxidative stress, which was effectively mitigated by selenium and vitamin E supplementation [15].

Selenium supplementation in patients with type 2 diabetes and Hashimoto’s thyroiditis improved the lipid profile, lowering LDL and triglyceride levels and increasing HDL levels. In addition, selenium contributed to better glycemic control, lowering HbA1c and fasting glucose levels. The results suggest that selenium may promote diabetes management by improving both glucose metabolism and lipid parameters [16]. Peruzzu et al. demonstrated that in patients with type 1 diabetes, serum selenium concentrations were significantly correlated with triglyceride levels in women (*p* = 0.0113). However, no significant associations were found between selenium and other lipid parameters (LDL, HDL, and total cholesterol) or glycemic control markers such as HbA1c and fasting glucose. These results suggest that selenium has a limited but specific role in lipid metabolism, particularly in female patients with T1DM [17].

Age is one of the key determinants of metabolic health, as it has been strongly associated with increased insulin resistance, elevated glucose levels, and adverse changes in lipid profiles. In a 10-year follow-up study among Chinese adults, Zhao et al. (2020) demonstrated that aging significantly increases the risk of developing metabolic syndrome, primarily through its effects on fasting glucose, triglycerides, and HDL cholesterol levels [18].

In the present study, serum selenium concentration and age were conceptualized as independent variables to examine their predictive value for markers of lipid and carbohydrate metabolism.

Although direct studies on the moderating role of HbA1c in the relationship between selenium status and lipid profile are limited, some evidence suggests that glycemic control may influence selenium–lipid interactions. HbA1c, as a stable marker of long-term glycemic status, could affect the biochemical effects of selenium on lipid parameters. Wu et al. demonstrated that genetically predicted selenium levels were positively associated with HbA1c and negatively associated with total and LDL cholesterol, implying that glycemic status may alter selenium–lipid relationships [19]. These findings support the rationale for examining HbA1c as a potential moderator in this context.

Based on this background, we formulated the following hypothesis: serum selenium concentration and age are significant predictors of selected lipid and glycemic markers in middle-aged women, and HbA1c moderates the relationship between selenium and lipid parameters, reflecting a potential interaction between glucose and lipid metabolism.

The aim of the study was to assess whether serum selenium concentration and age are significant predictors of markers of lipid and carbohydrate metabolism in middle-aged women. In addition, this study explored whether glycated hemoglobin (HbA1c) moderates the association between selenium and selected lipid parameters.

## 2. Materials and Methods

### 2.1. Organization and Course of the Study

This study was conducted in accordance with ethical standards and the Declaration of Helsinki. The study protocol was approved by the Bioethics Committee of the Pomeranian Medical University in Szczecin, Poland (no. KB-0012/181/13).

The study sample consisted of 387 middle-aged women living in northwestern Poland. Recruitment for the study was carried out through advertisements in the local press, as well as information posters and leaflets distributed in public places. Before starting the research, the respondents were informed about its course and purpose and the possibility of opting out at any stage. Each of the women gave informed written consent to participate in the study.

The following inclusion criteria were adopted:

- Female sex;
- No selenium supplementation;
- No history of inflammatory, psychiatric, or cancerous diseases;
- No alcohol abuse.

The norm for alcohol consumption was less than 20 g of pure alcohol a day or occasionally no more than 40 g of pure alcohol and at the declaration of at least two days of abstinence from alcohol a week (the State Agency for Solving Alcohol Problems) [20]. Women who did not meet all the above criteria were not included in the study. Laboratory data available from the parent database were reviewed to assess hemoglobin status. None of the women included in this study met the World Health Organization diagnostic criteria for anemia (hemoglobin < 12 g/dL) [21], and therefore, no participants with anemia were included in the final analysis.

This study consisted of three stages. The first stage involved a diagnostic survey performed using a questionnaire technique. The questionnaire concerned the following data:

- Sociodemographic data;
- Selected medical information on chronic diseases, medications taken for hypertension, hypertriglyceridemia, hyperglycemia, and elevated HDL levels;
- Addiction to cigarettes and alcohol.

In the second stage, a Tanita MC780 MA device (Tanita Corporation, Tokyo, Japan) was used to assess the subjects’ body composition. The mass of body fat (MBF) in kilograms and percentage of body fat (PBF), as well as the amount of visceral fat, were measured. The normal PBF for women aged 40–59 is 23–34%, and for women aged 60–79 is 24–36%. Normal values for visceral adipose tissue range from 1 to 12. Body weight and height were also measured. Body mass index (BMI) was calculated using the following formula: weight in kilograms divided by height in meters squared (kg/m^2^).

The following norms for BMI were adopted:

- The range of 18.5–24.9―normal weight;
- Below 18.5―underweight;
- The range of 25.0–29.9―overweight;
- Above 30.0―obesity.

In the third stage of the study, biological material (blood) was collected from a venous vessel in accordance with the procedure for collecting, storing, and transporting biological material from a peripheral vein. BD Vacutainer plastic tubes (Sarstedt, Nümbrecht, Germany) were used to collect the material. Blood was collected between 7.00 and 9.30 in the morning after an overnight fast of about 8–12 h and 10 min OF rest in a sitting position just before collecting the material for analysis.

Serum selenium concentrations were measured (levels between 93 and 121 µg/L were regarded acceptable). The content of selenium was determined by the spectrofluorimetric method using 2,3-diaminonaphthalene (the Shimadzu RF-5001 PC). The samples were wet-digested in a mixture of concentrated acids HNO_3_ (230 °C, 180 min) and HClO_4_ (310 °C, 20 min). The measurement was performed at the emission wavelength of 518 nm and the excitation wavelength of 378 nm. To ensure the accuracy and reproducibility of the selenium assay, internal quality control (IQC) and external quality assurance (EQA) procedures were implemented. The method was validated through the evaluation of the detection limit (LOD), quantification limit (LOQ), analyte recovery (ranging from 95 to 105%), and linearity, with the results verified using certified reference materials (e.g., NIST SRM 1577c Bovine Liver). Additionally, the laboratory participated in interlaboratory comparison programs, ensuring the precision and comparability of the determinations.

To diagnose carbohydrate metabolism disorders, venous serum glucose, insulin, and glycated hemoglobin (HbA1c) levels were determined, and the HOMA-IR index was calculated.

We adopted the following reference values according to the 2022 position of the Polish Diabetes Association:

Fasting glucose values are given as follows:

- Values < 70 mg/dL―hypoglycemia;
- Values 70–99 mg/dL―normal fasting glucose;
- Values 100–125 mg/dL (5.6–6.9 mmol/dL)―impaired fasting glucose (IFG);
- Values > 125 mg/dL―diabetes.

The norm for a fasting insulin level is 5–12 µU/mL. The HbA1c value reflects the average blood glucose concentration over a period of about three months preceding the test, with about 50% of the HbA1c present in the blood formed in the last month before the test.

Fasting HbA1c values are given as follows:

- HbA1c ≤ 6.5%―normal value;
- HbA1c > 6.5%―diabetes.

The Homeostasis Model Assessment–Insulin Resistance (HOMA-IR) method is a validated tool used to quantify insulin resistance (IR) based on fasting blood glucose and insulin concentrations. It is calculated according to the following formula: insulin (mU/mL) × glucose (mg/dL)/405 [22]. HOMA-IR values are given as follows:

- HOMA-IR < 2.5―normal value;
- HOMA-IR ≥ 2.5―insulin resistance.

For a broader picture of the subject’s health status, LDL cholesterol [mg/dL], HDL cholesterol [mg/dL], and triglycerides (TGs) [mg/dL] were also determined. The following values were regarded as abnormal:

- Serum triglycerides ≥ 150 mg/dL;
- HDL cholesterol (HDL-C) ≤ 50 mg/dL;
- LDL cholesterol (LDL-C) ≥ 100 mg/dL.

### 2.2. Statistical Analysis

In the statistical analysis, descriptive methods were used to present data for individual variables.

The normality of the data distribution was assessed using the Shapiro–Wilk test, which is recommended for smaller samples due to its high statistical power. In cases where the test indicated potential deviations from normality (*p* < 0.05), skewness was additionally tested. If the value of absolute skewness exceeded ±1, suggesting significant asymmetry, a Box–Cox transformation was applied to improve the normality of the data. After the transformation, normality was reassessed. Statistical analyses were performed on both the raw data and, if necessary, the transformed data.

The mean and standard deviation were calculated for quantitative variables, along with the frequency and percentage for categorical variables. To compare selenium concentrations between groups with different levels of fasting glucose, insulin, HbA1c, and HOMA-IR, Student’s *t*-test was applied, with the effect size estimated using Cohen’s d coefficient. Linear regression analysis was performed to examine the relationship between selenium concentration and other variables.

Sample Size and Power Calculation: We conducted an a priori power analysis for the planned multiple linear regression (with serum selenium, age, and their interactions as predictors) using GPower 3.1. We assumed a small effect size for the interaction term (Cohen’s f^2^ = 0.02, corresponding to ~2% of the variance explained) in the absence of prior data on the selenium × age effect. With α = 0.05 and power (1 − β) = 0.80, the analysis indicated that a minimum sample of ~395 subjects would be required to detect the interaction. Our final sample size of 387 participants is very close to this target, providing approximately 78–80% power for detecting the hypothesized interaction effect. Notably, this sample size also provides >90% power for a moderate interaction effect (f^2^ in the 0.10–0.15 range). We, therefore, consider our study adequately equipped to examine the influence of the selenium-by-age interaction on metabolic outcome markers. Power analysis was performed using GPower 3.1, following Faul et al. (2007) [23].

All calculations were performed using STATISTICA™ 13.3 software (TIBCO Software, Palo Alto, CA, USA). For all analyses, the *p*-level of <0.05 was considered statistically significant.

## 3. Results

The study included 387 women, of which 56.59% had tertiary education, and 36.69% had secondary education, 78.55% came from cities with over 100,000 inhabitants, 73.90% were in a formal relationship, and 90.70% were employed. Overweight was observed in 41.09% of participants, 19.12% were obese, and 39.53% had normal weight. Most of the respondents were non-smokers (65.63%) (Table 1).

The mean serum selenium concentration was 99.33 ± 19.79 µg/L. The mean values for selenium in the study group were within normal limits. The mean age was 52.55 ± 5.02 years. The mean distribution of individual anthropometric parameters was the following: 72.20 ± 13.55 kg for weight, 163.45 ± 6.70 for height, 26.56 ± 4.57 kg/m^2^ for BMI, 24.41 ± 8.48 kg for the mean fat mass, 32.93 ± 5.71% for the mean fat percentage, and 7.06 ± 2.40 for the mean visceral fat. The average value for BMI in the study group was above the normal limit. The other results of anthropometrics measurements (MBF, PBF, and visceral adipose tissue) were within the limits of the norm. The mean values of diabetes and insulin resistance markers were as follows: 85.60 ± 12.43 mg/dL for fasting glucose levels, 9.79 ± 5.60 µU/mL for insulin, and 5.41 ± 0.45% for HbA1c. The mean HOMA-IR index was 2.11 ± 1.35. The mean results for diabetes markers in the study group were within normal limits. The mean HDL cholesterol level was 66.91 ± 16.40 mg/dL, the LDL cholesterol level was 123 ± 32.50, and the triglyceride level was 107.09 ± 54.77 mg/dL. The mean results for HDL cholesterol were above the normal limits; triglycerides were below the normal limits in the study group. The mean results for LDL cholesterol were within the normal limits in the study group (Table 2).

The study group was also characterized in terms of markers of diabetes and insulin resistance. Less than 8% of the subjects had abnormal fasting glycemia (≥100 mg/dL), and about 3% had fasting insulin levels ≥25 µU/mL. Less than 2% had HbA1c (%) ≥ 6.5%, 25.52% had HOMA-IR ≥ 2.5, and about 1% had fasting blood glucose ≥ 126 mg/dL (Table 3).

We analyzed the effect of the serum selenium concentration on individual markers of diabetes and insulin resistance. No relationship between serum selenium concentration and individual markers of diabetes and insulin resistance was observed (*p* > 0.05) (Table 4).

A linear regression analysis was conducted to examine whether the selenium concentration (Se, measured in µg/L) or age (in years) predicted levels of high-density lipoprotein cholesterol (HDL-C, measured in mg/dL). The model was not statistically significant (*F*(2, 384) = 2.53, *p* = 0.08) and explained a negligible proportion of the variance in HDL-C, as indicated by an adjusted R^2^ of 0.01 (Table 5).

Despite the overall non-significant model, selenium concentration emerged as a significant predictor of HDL-C levels. Specifically, higher selenium concentrations were associated with higher HDL-C values (B = 0.09, SE = 0.04), with this effect reaching statistical significance (*t* = 2.23, *p* = 0.03). The standardized coefficient for selenium was β = 0.11, and the 95% confidence interval for the unstandardized estimate ranged from 0.01 to 0.22, suggesting a small but reliable positive effect.

In contrast, age did not significantly predict HDL-C levels (*t* = −0.05, *p* = 0.96), with a negligible standardized coefficient (β = −0.002) and a 95% confidence interval for the unstandardized estimate spanning from −0.10 to 0.10.

Overall, while the regression model did not reach statistical significance at the model level, selenium concentration independently predicted HDL-C levels, indicating a potential link worth exploring in future research. However, it is important to note that the proportion of variance explained by the model was minimal (adjusted R^2^ = 0.01), which limits the clinical relevance of this association despite its statistical significance (Table 6).

Summary: The selenium concentration was a statistically significant predictor of HDL-C levels, whereas age showed no significant effect.

A linear regression analysis was performed to investigate whether selenium concentration (Se, in µg/L) and age predict levels of low-density lipoprotein cholesterol (LDL-C, in mg/dL). The overall model was statistically significant (*F*(2, 384) = 5.95, *p* = 0.003) and accounted for a small portion of the variance in LDL-C, as indicated by an adjusted R^2^ of 0.03 (Table 7).

Within the model, age was a significant predictor of LDL-C levels. Specifically, a higher age was associated with higher LDL-C concentrations (B = 0.91, SE = 0.33), and this relationship was statistically significant (*t* = 2.75, *p* = 0.006). The standardized coefficient for age was β = 0.14, with a 95% confidence interval for the unstandardized coefficient ranging from 0.04 to 0.24.

In contrast, selenium concentration did not significantly predict LDL-C levels (B = 0.13, SE = 0.08), with the effect of failing to reach significance (*t* = 1.59, *p* = 0.112). The standardized coefficient for selenium was β = 0.08, and the corresponding 95% confidence interval ranged from −0.02 to 0.18, indicating that the observed effect could plausibly be zero.

These results suggest that while the regression model was statistically significant, the predictive power was modest and driven primarily by the effect of age on LDL-C levels rather than by selenium concentration (Table 8).

Summary: Age was a significant predictor of LDL-C levels, while selenium concentration was not associated with LDL-C in this model.

Neither selenium nor age significantly predicted triglyceride levels (Supplementary Tables S1 and S2).

A linear regression analysis was conducted to determine whether selenium concentration (Se, in µg/L) and age predict fasting glucose levels (in mg/dL). The overall model was statistically significant (*F*(2, 384) = 7.64, *p* < 0.001) and accounted for a small but non-negligible proportion of variance in glucose levels, with an adjusted R^2^ of 0.03 (Table 9).

Within the model, age emerged as a significant predictor of glucose levels. Specifically, higher age was associated with higher fasting glucose (B = 0.49, SE = 0.13), and this effect was statistically significant (*t* = 3.88, *p* < 0.001). The standardized regression coefficient was β = 0.20, and the 95% confidence interval for the unstandardized coefficient ranged from 0.10 to 0.30, indicating a robust positive relationship.

In contrast, selenium concentration did not significantly predict glucose levels. The regression coefficient for selenium was small and non-significant (B = −0.005, SE = 0.03) with *t* = −0.15, *p* = 0.881, and a standardized coefficient β = −0.01. The 95% confidence interval for this effect ranged from −0.11 to 0.09, suggesting that the relationship between selenium and glucose levels is likely negligible in this sample.

These results indicate that age is a significant positive predictor of fasting glucose, while selenium concentration does not appear to be associated with glucose levels (Table 10).

Summary: Age significantly predicted fasting glucose levels, while selenium had no measurable effect in this model.

The linear regression model evaluating the predictive value of selenium concentration and age for fasting insulin levels was not statistically significant, and neither variable showed a meaningful association with insulin. Detailed results are presented in Supplementary Tables S3 and S4.

A linear regression analysis was conducted to investigate whether selenium concentration (Se, in µg/L) and age predict hemoglobin A1c levels (HbA1c, in %). The overall model was statistically significant (*F*(2, 384) = 8.45, *p* < 0.001), explaining approximately 4% of the variance in HbA1c levels, as indicated by an adjusted *R*^2^ of 0.04 (Table 11).

Among the predictors, age was a significant positive predictor of HbA1c. Specifically, greater age was associated with higher HbA1c levels (B = 0.02, SE = 0.005), and this effect was statistically significant (*t* = 4.10, *p* < 0.001). The standardized regression coefficient was β = 0.21, with a 95% confidence interval for the unstandardized coefficient ranging from 0.11 to 0.31, indicating a reliable and meaningful association.

In contrast, selenium concentration did not significantly predict HbA1c levels. The regression coefficient for selenium was B = −0.001 (SE = 0.001), with a non-significant *t* value of −1.03 (*p* = 0.306) and a standardized coefficient of β = −0.05. The 95% confidence interval ranged from −0.15 to 0.05, suggesting the absence of a substantive effect.

In summary, the regression model identified age as a significant predictor of HbA1c, while the selenium concentration did not contribute significantly to the prediction of glycemic control in this sample (Table 12).

Summary: Age was a significant predictor of HbA1c levels, whereas selenium concentration was not statistically significant.

The interaction between selenium concentration and age in predicting HbA1c levels was examined using moderation analysis. The interaction effect was not statistically significant, indicating that age did not moderate the relationship between selenium and HbA1c. The full results are presented in Supplementary Tables S5 and S6.

The regression model evaluating the effect of selenium concentration and age on HOMA-IR did not yield statistically significant results. Neither predictor contributed meaningfully to the variance in insulin resistance in this sample. Detailed findings are available in Supplementary Tables S7 and S8.

## 4. Discussion

Selenium is an essential trace element involved in selenoprotein synthesis, contributing to antioxidant defense and cytoprotection [24]. While oxidative stress has been implicated in the development of type 2 diabetes [25], the relationship between selenium concentration and diabetes remains unclear. Some studies suggest that selenium-dependent glutathione peroxidases (GPxs) play a role in oxidative stress regulation, yet findings are inconsistent. De Vega et al. reported decreased GPx activity and lower selenium concentrations in diabetic patients, but the causal link remains debatable [26].

While some studies suggest a potential link between selenium concentration and diabetes risk [27], our results did not confirm such an association. This discrepancy may stem from differences in the study design, population characteristics, and selenium intake levels.

In our study, the mean serum selenium concentration was within normal limits. Winther et al. found that dietary selenium intake depends on geographic location [28]. In The European Prospective Investigation into Cancer and Nutrition (EPIC) conducted in ten European countries, the mean serum selenium concentration was 85.6 μg/L [29], which was a below-normal result. Both the lowest and the highest intake were observed in China, where the plasma selenium concentration ranged from 22 to 550 μg/L [30,31]. High selenium intake has been noted in North America—the mean serum selenium concentration in US residents was 137 μg/L [32]. Based on available studies, the mean serum selenium concentration in healthy women in Poland was approximately 70.4 ± 14.7 μg/L [33].

The results of clinical trials performed by Jacobs et al. did not confirm the causal role of selenium in the development of insulin resistance or type 2 diabetes, as no significant differences in β-cell function (HOMA2-%β) and insulin sensitivity (HOMA2-%S) were observed between individuals receiving selenium supplementation (200 μg/day) and those in the placebo group over a 2.9-year period. Additionally, fasting blood glucose levels were higher in the placebo group than in the selenium group. The authors suggested that individual characteristics, such as genotypes, may influence the metabolic response to selenium [22]. Similarly, a study conducted by Gao et al. in Sweden did not support the role of selenium in the development of glucose metabolism disorders or diabetes in the elderly [34]. Moreover, findings from the Selenium and Vitamin E Cancer Prevention Trial (SELECT) and other studies indicated no increased risk of type 2 diabetes following selenium supplementation at 200 μg/day compared to the placebo [35]. These results align with our findings, which did not establish a significant association between selenium concentration and diabetes markers.

In our own study on a group of women, no significant correlations were found between the serum selenium concentration and markers of diabetes and insulin resistance, such as fasting glucose, insulin, HbA1c, and HOMA-IR. However, recent evidence suggests that other molecular mediators, such as adiponectin, may play a key role in the relationship between selenium status and glucose metabolism. Adiponectin is an adipokine with anti-inflammatory and insulin-sensitizing properties, and its deficiency has been linked to the pathogenesis of insulin resistance and type 2 diabetes. It may represent the missing link in the selenium–glucose pathway, especially under conditions of oxidative stress or metabolic dysfunction. As highlighted in a comprehensive review by Begum et al., adiponectin is increasingly recognized as a promising target in the management of diabetes and its complications [36]. However, a positive correlation was observed between the serum selenium concentration, HDL cholesterol levels, and the age of the subjects. Although a positive association was found between selenium concentration and HDL-C levels, it should be emphasized that HDL-C concentration does not necessarily reflect HDL functionality, especially in postmenopausal women. During the menopausal transition, HDL particles undergo compositional and structural changes that may impair their cardioprotective capacity despite preserved or even increased HDL-C levels. This has been demonstrated in the SWAN-HDL study, where the HDL function (measured as cholesterol efflux capacity) decreased across menopause, even as HDL-C increased [37]. This age-related increase in cholesterol may reflect not only biological aging but also the menopausal transition. It is well documented that total and LDL cholesterol levels rise significantly after menopause, primarily due to decreased estrogen levels [38,39]. Since menopausal status was not assessed in the present study, it is possible that some of the associations observed between age and lipid parameters are partly mediated by menopausal changes rather than age alone.

While HDL cholesterol was found to be a significant predictor of selenium concentration, exploratory subgroup patterns suggested that the relationship between selenium and lipid markers (HDL and LDL) may differ depending on HbA1c levels. In particular, we observed that the positive association between selenium and HDL cholesterol appeared stronger among participants with elevated HbA1c values. This may reflect potential interactions between glycemic control and lipid metabolism. However, as no formal moderation analysis (e.g., interaction terms in regression) was performed, these findings should be interpreted cautiously. Future studies should explore these relationships using appropriate statistical models to test moderation effects.

A study by Hwang et al. conducted on non-obese diabetic mice given 0.2 mol/L Se in drinking water showed lower serum glucose levels and better lipid metabolism than before supplementation [40]. In their study conducted in a group of over 7000 subjects, Park et al. analyzed the long-term effects of selenium exposure and the risk of type 2 diabetes in healthy individuals. They found that those with the highest levels of selenium in their toenails had a 24% lower risk of developing diabetes than those with a lower selenium concentration [29]. However, our study did not confirm such an association, which may be due to differences in the study design, selenium exposure levels, or population characteristics.

Fontenelle et al. reviewed the existing literature and suggested that selenium may be involved in the regulation of insulin signaling and carbohydrate metabolism in the liver. They proposed that both selenium deficiency and excess could influence insulin resistance, indicating a potential optimal concentration range for metabolic health [41]. However, these findings remain debated. Similarly, Siddiqi et al. observed an inverse relationship between selenium intake and type 2 diabetes risk in a northern Chinese population, where higher selenium intake was associated with a lower risk of the disease [42]. In contrast, our study did not confirm a significant association between selenium concentration and diabetes markers. Meanwhile, Raygan et al. reported that selenium supplementation for 12 weeks in patients with congestive heart failure resulted in improved lipid profiles, including reduced total and LDL cholesterol and increased HDL cholesterol [43]. This is consistent with our findings regarding selenium’s positive correlation with HDL cholesterol levels.

Our findings are partially consistent with some scientific reports. The lack of a significant relationship between the selenium concentration and markers of diabetes and insulin resistance is consistent with the results of Jacobs et al. [22]. However, other studies, such as those by Fontenelle et al. [41] and Siddiqi et al. [42], suggest a beneficial effect of selenium on insulin sensitivity and a lower risk of type 2 diabetes. The positive correlation between selenium concentration and HDL levels observed in the study is consistent with the results of Raygan et al. [43], who also found a beneficial effect of selenium supplementation on lipid profiles. Differences in the results may be due to different study populations, selenium doses, forms of supplementation, and study methodologies. It is noteworthy that some studies outline both the beneficial and neutral effects of selenium on glucose and lipid metabolism, indicating the need for further research to clearly define the role of this element in these processes.

Limitations: The sample size (n = 387) was selected on the basis of the available population and ethical considerations, which limited the ability to use rigorous sample size planning methods before the study began. No formal a priori power analysis was performed, but the post hoc analysis conducted showed that the study had sufficient power (>80%) to detect moderate-sized effects.

This study was conducted only on middle-aged women, which limits the generalizability of the results to the male population and people in other age groups. The results may vary depending on gender, age, and other demographic factors.

In addition, due to the cross-sectional nature of the study, the causal relationship between serum selenium concentration, diabetes markers, and lipids cannot be clearly determined. Further studies are needed to confirm or exclude the long-term effect of selenium.

It is also worth noting that selenium’s concentration in the body is strongly dependent on diet and supplementation. The lack of precise data on the dietary intake of selenium in the women studied limits the interpretation of the results.

In our study, selenium intake from the diet was not directly assessed, which is a limitation. However, we measured serum selenium concentrations, which allowed us to assess the selenium concentration in the body. Diet is the main source of selenium, and its content in food depends on the concentration of this element in the soil and the dietary habits of the population. Future studies should include a detailed dietary assessment to better understand the effects of selenium intake on glucose metabolism and the risk of developing diabetes.

This study did not take into account other factors that can affect selenium concentration, such as heavy metal exposure, thyroid functional status, and inflammation and oxidative stress levels, among others.

The way selenium is metabolized and its effect on glucose and lipid metabolism may depend on genetic determinants. The lack of analysis of polymorphisms of genes related to selenium metabolism (e.g., SELENOP) may mean that individual differences in response to selenium have not been accounted for.

Although blood selenium concentrations were determined, the activity of selenoproteins, such as glutathione peroxidases (GPxs), which play a key role in antioxidant defense, was not assessed. This may limit the interpretation of the results in the context of selenium’s effects on oxidative stress and metabolism. Most of the participants had right-leaning levels of glucose, HbA1c, and HOMA-IR, which may limit their ability to detect significant relationships in a population at higher risk for type 2 diabetes.

Further prospective and experimental studies are needed to better understand the role of selenium in regulating lipid and glucose metabolism.

Moreover, this study did not include several important confounding factors that could influence both selenium status and metabolic health. These include dietary selenium intake, physical activity levels, detailed socioeconomic status indicators (beyond basic education and employment categories), and thyroid function. While the serum selenium concentration was directly measured, it may not fully reflect recent intake, bioavailability, or individual variability in absorption. Likewise, both thyroid dysfunction and low physical activity are known to affect lipid and glucose metabolism and may confound the observed associations. The absence of these variables may have introduced residual confounding, and thus, the findings should be interpreted with appropriate caution. Future studies should aim to incorporate these factors to provide a more comprehensive understanding of the relationship between selenium status and metabolic markers. Although BMI was assessed in this study, it was not included as a covariate in the regression models, which may have influenced the observed associations. Notably, similar methodological approaches have been applied in recent large-scale studies examining selenium and metabolic outcomes, where BMI was not adjusted for in multivariate analysis despite its known metabolic relevance [44].

Future studies should consider using more advanced statistical approaches, including stepwise regression and formal moderation or mediation models, to better capture complex interactions between selenium, glycemic markers, and lipid parameters.

Implications: Because of selenium’s possible effects on the lipid profile and glucose metabolism, it is worth including selenium labeling in the routine diagnosis of patients with metabolic disorders. Our findings suggest that selenium supplementation should be individually tailored, especially in patients with carbon-date disorders, to avoid potentially adverse metabolic effects. Patients with elevated HbA1c levels and abnormal lipid profiles should be educated about the possible interactions between selenium concentration and lipid and glucose metabolism.

There is a need for prospective studies to confirm the effects of selenium on metabolism, which may help to better tailor dietary and supplementation strategies.

## 5. Conclusions

Age consistently emerged as a significant predictor in most of the analyzed models. In particular, a significant positive association with age was observed for low-density lipoprotein cholesterol (LDL-C), fasting glucose, and glycated hemoglobin (HbA1c), indicating that the risk of adverse changes in metabolic parameters related to lipid and carbohydrate metabolism increases with age. In the models concerning insulin and HOMA-R, age did not reach statistical significance, although, in the case of HOMA-R, a trend was observed that may indicate a potential association.

In contrast to age, selenium concentration demonstrated only limited predictive value. It was a statistically significant positive predictor only for HDL cholesterol, which may suggest that there is a beneficial effect of selenium on the lipid profile; however, this effect was small. In all other models (LDL, TG, glucose, insulin, HbA1c, and HOMA-R), selenium was not a significant predictor, and its effects fell within confidence intervals that included zero, indicating a lack of clear evidence for its influence on these parameters in the analyzed sample.

## Figures and Tables

**Table 1:** Characteristics of the study sample―sociodemographic data.

Variables		n	%
Education	primary	3	0.78
	vocational	23	5.94
	secondary	142	36.69
	tertiary	219	56.59
Place of residence	rural area	36	9.30
	city of up to 10,000 inhabitants	10	2.58
	city of up to 100,000 inhabitants	37	9.56
	city of over 100,000 inhabitants	304	78.55
Marital status	single	59	15.25
	informal relationship	42	10.85
	formal relationship	286	73.90
Employment status	unemployed	36	9.30
	employed	351	90.70
BMI category	underweight (<18.50)	1	0.26
	normal weight (18.50–25.00)	153	39.53
	overweight (25.00–29.99)	159	41.09
	obesity (≥30.00)	74	19.12
Smoking	yes	133	34.37
	no	254	65.63

**Table 2:** Characteristics of the study sample.

Parameter		M	SD	Min	Max	CV [%]
Se [mg/L]		99.33	19.79	53.16	182.00	19.92
Age [years]		52.55	5.02	43.00	67.00	9.56
Weight [kg]		72.20	13.55	42.50	128.40	18.77
Height [cm]		163.45	6.70	138.9	194.0	4.09
BMI [kg/m^2^]		26.56	4.57	17.50	42.90	17.21
MBF [kg]		24.41	8.48	9.20	63.20	34.73
PBF [%]		32.93	5.71	16.50	49.20	17.33
Visceral adipose tissue		7.06	2.40	3.00	15.00	34.03
Diabetes markers	Norm					
Fasting glucose [mg/dL]	70–99 mg/dL	85.60	12.43	9.70	185.30	14.52
Insulin [µU/mL]	5–12 μIU/mL	9.79	5.60	1.80	33.90	57.14
HbA1c (%)	≤6.5 mg/dL	5.41	0.45	4.58	10.94	8.38
HOMA-IR	<2.5	2.11	1.35	0.24	7.72	64.00
Lipid profile	Norm					
HDL cholesterol [mg/dL]	≤50 mg/dL	66.61	15.79	28.50	117.60	23.71
LDL cholesterol [mg/dL]	≥100 mg/dL	123.75	32.50	11.28	223.18	26.26
Triglycerides [mg/dL]	≥150 mg/dL	107.09	54.77	35.10	379.60	51.14

M—mean; SD—standard deviation; CV—coefficient of variation; Min—minimum value; Max—maximum value; MBF—mass of body fat; PBF—percentage of body fat.

**Table 3:** Characteristics of the study sample with regard to markers of type 2 diabetes.

Variables	Type 2 Diabetes Markers	n	%
Fasting glucose [mg/dL]	[≥100 mg/dL]	30	7.75
Insulin [µU/mL]	[≥25 µU/mL]	12	3.10
HbA1c (%)	[≥6.5%]	6	1.55
HOMA-IR	[≥2.5]	98	25.32

**Table 4:** Comparative analysis of the mean serum selenium concentration [mg/L] using particular markers of type 2 diabetes and IR.

Markers of Type 2 Diabetes and IR	Se [mg/L]		t_df=385_	*p*-Value *	D ** (95%CI)
	M	SD			
Fasting glycemia [≥100 mg/dL or pharmacotherapy for diabetes]; n = 30	99.55	20.47	0.062	0.951	−0.012 (−0.385; 0.360)
Fasting glycemia [<100 mg/dL]; n = 357	99.31	19.76			
Insulin [≥25 µU/mL]; n = 12	102.07	20.54	0.487	0.627	−0.143 (−0.718; 0.432)
Insulin [<25 µU/mL]; n = 375	99.24	19.79			
HbA1c (%) [≥6.5%]; n = 6	97.12	36.24	−0.275	0.783	0.114 (−0.693; 0.920)
HbA1c (%) [<6.5%−normal value]; n = 381	99.37	19.50			
HOMA-IR [≥2.5]; n = 98	101.07	20.22	1.005	0.315	−0.118 (−0.347; 0.112)
HOMA-IR [<2.5]; n = 289	98.74	19.64			

M—mean; SD—standard deviation; CI—confidence interval; * two-tailed Student’s *t*-test; ** Cohen’s *d* coefficient.

**Table 5:** Model fit summary for HDL cholesterol—linear regression results.

Model	Adjusted R^2^	F	df1	df2	*p*
1	0.01	2.53	2	384	0.08

**Table 6:** Regression coefficients for predictors of HDL cholesterol (selenium and age).

Predictor	Estimate	SE	*t*	*p*	Stand. Estimate	95% CI Lower	95% CI Upper
Intercept	57.93	8.82	6.57	<0.001	—	—	—
Se (µg/L)	0.09	0.04	2.23	0.03	0.11	0.01	0.22
Age	−0.01	0.16	−0.05	0.96	−0.00	−0.10	0.10

**Table 7:** Model fit summary for LDL cholesterol—linear regression results.

Model	Adjusted R^2^	F	df1	df2	*p*
1	0.03	5.95	2	384	0.00

**Table 8:** Regression coefficients for predictors of LDL cholesterol (selenium and age).

Predictor	Estimate	SE	*t*	*p*	Stand. Estimate	95% CI Lower	95% CI Upper
Intercept	62.84	18.00	3.49	<0.001	—	—	—
Se (µg/L)	0.13	0.08	1.59	0.11	0.08	−0.02	0.18
Age	0.91	0.33	2.75	0.01	0.14	0.04	0.24

**Table 9:** Model fit summary for glucose—linear regression results.

Model	Adjusted R^2^	F	df1	df2	*p*
1	0.03	7.64	2	384	<0.001

**Table 10:** Regression coefficients for predictors of glucose (selenium and age).

Predictor	Estimate	SE	*t*	*p*	Stand. Estimate	95% CI Lower	95% CI Upper
Intercept	60.48	6.86	8.82	<0.001	—	—	—
Se (µg/L)	−0.00	0.03	−0.15	0.88	−0.01	−0.11	0.09
Age	0.49	0.13	3.88	<0.001	0.20	0.10	0.30

**Table 11:** Model fit summary for hemoglobin A1C—linear regression results.

Model	Adjusted R^2^	F	df1	df2	*p*
1	0.04	8.45	2	384	<0.001

**Table 12:** Regression coefficients for predictors of hemoglobin A1C (selenium and age).

Predictor	Estimate	SE	*t*	*p*	Stand. Estimate	95% CI Lower	95% CI Upper
Intercept	4.54	0.25	18.22	<0.001	—	—	—
Se (µg/L)	−0.00	0.00	−1.03	0.31	−0.05	−0.15	0.05
Age	0.02	0.00	4.10	<0.001	0.21	0.11	0.31

## Data Availability

The dataset is available upon request from the corresponding author at the following email address: anna.cybulska@pum.edu.pl.

## Supplementary Materials

The following supporting information can be downloaded at: https://www.mdpi.com/article/10.3390/nu17091429/s1, Table S1: Model Fit Summary for Triglycerides—Linear Regression Results; Table S2: Regression Coefficients for Predictors of Triglycerides (Selenium and Age); Table S3: Model Fit Summary for Insulin—Linear Regression Results; Table S4: Regression Coefficients for Predictors of Insulin (Selenium and Age); Table S5: Moderation Estimates; Table S6: Simple Slope Analysis; Table S7: Model Fit Summary for HOMA-R—Linear Regression Results; Table S8: Regression Coefficients for Predictors of HOMA-R (Selenium and Age).
