# Supplementary material for: Serum Selenium and Age as Predictors of Metabolic Health in Middle-Aged Women: A Regression-Based Study
Source: Nutrients. 2025 Apr 24;17(9):1429. doi: 10.3390/nu17091429 (PMC12073846; doi:10.3390/nu17091429)
Supplement: Supplementary file 1 [file nutrients-17-01429-s001.zip › nutrients-3589311-supplementary.pdf]

Supplementary materials

These findings indicate that neither selenium concentration nor age provides a meaningful contribution to explaining variability in triglyceride levels within the studied sample (Supplementary Table S1, S2).

Summary: The model did not identify any significant predictors of triglyceride levels; both selenium and age were non-significant.

Supplementary Table S1. Model Fit Summary for Triglycerides – Linear Regression Results.

Model	Adjusted R^2	F	df1	df2	p
1.00	-0.00	0.61	2.00	384.00	0.54

Supplementary Table S2. Regression Coefficients for Predictors of Triglycerides (Selenium and Age).

Predictor	Estimate	SE	t	p	Stand. Estimate	95% CI Lower	95% CI Upper
Intercept	73.28	30.75	2.38	0.02	—	—	—
Se (μg/l)	0.06	0.14	0.41	0.68	0.02	-0.08	0.12
Age	0.53	0.56	0.94	0.35	0.05	-0.05	0.15

These findings indicate that, within this sample, neither selenium concentration nor age was associated with insulin levels, and the regression model as a whole lacked explanatory power (Supplementary Table S3, S4).

Summary: No significant predictors of insulin levels were identified; both selenium and age were non-significant.

Supplementary Table S3. Model Fit Summary for Insulin – Linear Regression Results.

Model	Adjusted R^2	F	df1	df2	p
1	-0.00	0.68	2	384	0.51

Supplementary Table S4. Regression Coefficients for Predictors of Insulin (Selenium and Age).

Predictor	Estimate	SE	t	p	Stand. Estimate	95% CI Lower	95% CI Upper
Intercept	6.26	3.14	1.99	0.05	—	—	—
Se (μg/l)	0.01	0.01	0.63	0.53	0.03	-0.07	0.13
Wiek	0.05	0.06	0.87	0.39	0.04	-0.06	0.15

A moderation analysis was performed to evaluate whether age moderates the relationship between selenium concentration (Se, in µg/l) and hemoglobin A1c levels (HbA1c, in %). The interaction between selenium and age was not statistically significant (Estimate = 0.00012, SE = 0.00024, Z = 0.51, p = .610), indicating that the relationship between selenium and HbA1c does not vary significantly across age levels.

In summary, while age remains a robust predictor of increased HbA1c, selenium concentration does not significantly predict HbA1c levels, nor is its effect moderated by age Supplementary Table S5, S6.

Supplementary Table S5. Moderation Estimates

	Estimate	SE	Lower	Upper	Z	p
Se (µg/l)	-0.00125	0.00114	-0.00349	9.92e-4	-1.092	0.275
Age	0.01884	0.00448	0.01005	0.0276	4.201	< .001
Se (µg/l) × Age	1.23e-4	2.42e-4	-3.51e-4	5.98e-4	0.510	0.610

Supplementary Table S6. Simple Slope Analysis

	Estimate	SE	Lower	Upper	Z	p
Average	-0.00125	0.00114	-0.00349	9.93e-4	-1.092	0.275
Low (−1SD)	-0.00187	0.00174	-0.00529	0.00155	-1.071	0.284
High (+1SD)	-6.29e–4	0.00159	-0.00374	0.00248	-0.396	0.692

Note. shows the effect of the predictor (Se [µg/l]) on the dependent variable (Hemoglobina A1C).

These findings suggest that neither selenium concentration nor age significantly contributes to explaining individual differences in HOMA-R values within this sample (Supplementary Table S7, S8).

Summary: No significant predictors of HOMA-IR were found; selenium and age did not show statistically significant associations.

Supplementary Table S7. Model Fit Summary for HOMA-R – Linear Regression Results.

Model	Adjusted R^2	F	df1	df2	p
1	0.01	2.15	2	384	0.12

Supplementary Table S8. Regression Coefficients for Predictors of HOMA-R (Selenium and Age)

Predictor	Estimate	SE	t	p	Stand. Estimate	95% CI Lower	95% CI Upper
Intercept	0.55	0.76	0.73	0.47	—	—	—
Se (µg/l)	0.00	0.00	0.54	0.59	0.03	-0.07	0.13
Age	0.03	0.01	1.88	0.06	0.10	-0.00	0.20
